# Use of RNA-Seq and a Transgenic Mouse Model to Identify Genes Which May Contribute to Mutant p53-Driven Prostate Cancer Initiation

**DOI:** 10.3390/biology11020218

**Published:** 2022-01-29

**Authors:** Ruth Vinall, Qian Chen, George Talbott, Rajendra Ramsamooj, An Dang, Clifford G. Tepper, Alexander Borowsky

**Affiliations:** 1 Department of Pharmaceutical & Biomedical Sciences, California Northstate University College of Pharmacy, Elk Grove, CA 95757, USA; gctalbott@gmail.com; 2 Department of Pathology, Davis School of Medicine, University of California, Sacramento, CA 95817, USA; qanchen@ucdavis.edu (Q.C.); andang@ucdavis.edu (A.D.); adborowsky@ucdavis.edu (A.B.); 3 Department of Clinical Medicine, California Northstate University College of Medicine, Elk Grove, CA 95757, USA; rajendra.ramsamooj@cnsu.edu; 4 Department of Biochemistry and Molecular Medicine, Davis School of Medicine, University of California, Sacramento, CA 95817, USA; cgtepper@ucdavis.edu

**Keywords:** prostate cancer, genetically engineered mouse model, mutant p53, RNA-Seq

## Abstract

**Simple Summary:**

We use RNA-seq analysis to identify genes that may contribute to mutant p53-mediated prostate cancer initiation in a genetically engineered mouse model (B6.129S4-Trp53tm3.1Tyj/J). A total of 1378 differentially expressed genes, including wildtype p53 target genes (e.g. Cdkn1a, Bax, Bcl2, Kras, Mdm2), p53 gain-of-function-related genes (Mgmt, Id4), and prostate cancer-related genes (Cav-1, Raf1, Kras), were identified. Mice that were homozygous or heterozygous for the Trp53 R270H mutation developed grade one PIN lesions at 3 months and 5 months, respectively, whereas wildtype mice did not develop PIN. Immunohistochemical analysis revealed decreased levels of irradiation-mediated apoptosis in homozygous and heterozygous mice when compared to wildtype counterparts, and this aligned with observed differences in apoptosis-related gene expression.

**Abstract:**

We previously demonstrated that the *Trp53-R270H* mutation can drive prostate cancer (CaP) initiation using the FVB.129S4 (Trp53^tm3Tyj/wt^); FVB.129S (Nkx3-1^tm3(cre)Mmswt^) genetically engineered mouse model (GEM). We now validate this finding in a different model (B6.129S4-*Trp53*^tm3.1Tyj^/J mice) and use RNA-sequencing (RNA-Seq) to identify genes which may contribute to *Trp53 R270H*-mediated prostate carcinogenesis. Wildtype (*Trp53^WT/WT^*), heterozygous (*Trp53^R270H/WT^*), and homozygous mice (*Trp53^R270H/R270H^*) were exposed to 5 Gy irradiation to activate and stabilize p53, and thereby enhance our ability to identify differences in transcriptional activity between the three groups of mice. Mouse prostates were harvested 6 h post-irradiation and processed for histological/immunohistochemistry (IHC) analysis or were snap-frozen for RNA extraction and transcriptome profiling. IHC analyses determined that presence of the *Trp53-R270H* mutation impacts apoptosis (lower caspase 3 activity) but not cell proliferation (Ki67). RNA-Seq analysis identified 1378 differentially expressed genes, including wildtype p53 target genes (E.g., *Cdkn1a*, *Bax*, *Bcl2*, *Kras*, *Mdm2*), p53 gain-of-function (GOF)-related genes (*Mgmt, Id4*), and CaP-related genes (*Cav-1, Raf1, Kras*). Further understanding the mechanisms which contribute to prostate carcinogenesis could allow for the development of improved preventive methods, diagnostics, and treatments for CaP.

## 1. Introduction

An estimated 1.5 million men are newly diagnosed with prostate cancer (CaP) each year (http://seer.cancer.gov/csr/ accessed on 13 December 2021). Understanding the mechanisms which contribute to CaP initiation is important because it can inform the development of chemoprevention strategies, which could reduce this number as well as support the development of improved diagnostic and prognostic biomarkers. CaP tumors are molecularly heterogeneous, indicating that CaP initiation can be caused by multiple different mechanisms [1,2]. In support of this, cell line and animal studies have identified several different genetic alterations which can independently play a causative role in CaP initiation. For example, loss of PTEN and amplification of Myc can both drive CaP initiation in mouse models [3]; these genetic alterations occur in up to 60 and 50% of CaP patients, respectively [4,5]. PTEN dysregulation promotes initiation by causing increased activity of the PI3K–Akt signaling pathway [3,6,7]. Amplification of Myc causes decreased Nkx3.1 expression and upregulation of PIM1 and EZH2, and thereby promotes increased cell plasticity [8]. Our group has previously demonstrated that the *Trp53-R270H* mutation can drive CaP initiation in FVB.129S4 (Trp53^tm3Tyj/wt^); FVB.129S (Nkx3-1^tm3(cre)Mmswt^) mice (Vinall et al., 2012). In this model, *Trp53^R270H/R270H^* mice developed prostatic intraepithelial neoplasia (PIN) as early as 6 weeks, and *Trp53^R270H/+^* mice developed PIN as early as 3 months [9]. CaP was detected at 8 months in *Trp53^R270H/R270H^* mice. It is estimated that ~30% of CaP patients with early stage disease harbor *Tp53* missense mutations, further supporting a role for p53 mutations in driving CaP initiation [10,11,12,13,14,15,16,17,18,19,20,21]. To our knowledge, the mechanisms by which p53 mutations drive CaP initiation remain completely unknown. 

Many of the *Tp53* missense mutations that are found in CaP patient tumors are gain-of-function (GOF) mutations, e.g., *Tp53 R273H*, *G245S*, *R273C*, and *R248W* (note that *Trp53-R270H* is the mouse equivalent of human *Tp53-R273H*). They are termed ‘GOF’ because the mutant proteins for which they encode can regulate the activity of genes that wildtype p53 does not usually regulate, e.g., *BCL-XL* and *MDR1* [22]. These GOF mutations are similar to other p53 missense mutations, in as much as they still confer loss-of-function (LOF); they are also unable to regulate, or unable to fully regulate, the expression of wildtype p53 target genes, e.g., *Cdkn1a*, *Bax*, and *Bbc3* [23]. This dual functionality that is displayed by *Tp53* GOF mutants has led some researchers to suggest that *Tp53* should be classified as both a proto-oncogene and tumor suppressor [24]. Multiple studies have investigated the role of p53 mutations in promoting CaP progression [23,25,26,27,28,29,30], and some of the underlying mechanisms by which *Tp53* GOF mutations drive CaP progression have been elucidated. For example, our group has demonstrated that the *Tp53 R273H* mutation can drive castration resistant (CR) growth of LNCaP cells via increasing the transcription of H2 relaxin, a peptide hormone [23]. Increased expression of H2 relaxin causes increased activity of the Akt and NFkappaB signaling pathways and thereby promotes cell proliferation [29,30]. It is possible that some of these mechanisms could also contribute to *Tp53* GOF mutant-driven CaP initiation.

The goal of the current study was to identify gene expression changes that may play a role in facilitating p53 GOF mutant-driven CaP initiation. A mouse model which harbors the *Trp53 R270H* mutation (B6.129S4-*Trp53*^tm3.1Tyj^/J) was used for these studies. Inclusion of mice that are heterozygous and homozygous for the *Trp53 R270H* mutation allowed for gene dosage effects to be determined and for parallels with early-stage human prostate carcinogenesis to be considered; the majority of CaP patients are heterozygous for *Tp53* mutations and loss of heterozygosity (LOH) typically only occurs during late disease. The use of RNA-seq analysis provided an unbiased approach. 

## 2. Materials and Methods

### 2.1. Mice

The *Trp53 R270H* mice (B6.129S4-*Trp53*^tm3.1Tyj^/J) used for this study were obtained from Jackson Labs (Bar Harbor, ME, USA). These mice were originally generated by the Tyler Jacks lab [31]. All animal procedures were reviewed and approved by the UC Davis Institutional Animal Care and Use Committee (IACUC, protocol approval # 18457). 

### 2.2. Breeding and Genotyping

Breeding and genotyping was performed as previously described [9]. The following primers were used to distinguish between wildtype, heterozygous, and homozygous mice: F- 5′-agctagccaccatggcttgagtaa gtctgca-3′; R- 3′-cttggagacatagccacactg-3′ [32]. Amplification of the wildtype allele yielded a 290 bp PCR product, while amplification of the mutant p53 allele yielded a 330 bp PCR product. 

### 2.3. Irradiation Studies

Mice that were ~3 months old were exposed to 5 Gy whole body irradiation (Shepard cesium source model 143-68 irradiator, San Fernando, CA, USA) to activate and stabilize p53, consequently increasing its expression. Mouse prostates were harvested 6 h post-irradiation and either processed for subsequent histological and/or immunohistochemistry (IHC) analysis or snap-frozen for subsequent RNA extraction and transcriptome profiling with RNA-Sequencing (RNA-Seq) analysis (please see below for details). 

### 2.4. Histology and Immunohistochemistry

Standard histopathology and IHC was performed as described previously [33]. The presence and number of PIN lesions was assessed by the pathological analysis of hematoxylin and eosin (H&E)-stained sections. Primary antibodies used for IHC analyses were p53 (1:1000: Santa Cruz Biotech Inc., Santa Cruz, CA, USA), Ki67 Ab-4 (1:500; Neomarker, Fremont, CA, USA), and activated caspase 3 (1:1000; Promega, Madison, WI, USA). For the assessment of activated caspase 3 and Ki67 positivity, the total number of prostate epithelial cells versus the number of positive cells was assessed in 3 randomly selected fields of view (×20 magnification) and the percentage of positivity was calculated. 

### 2.5. RNA Isolation and Sequencing Analysis

Samples were submitted to the UC Davis Comprehensive Cancer Center’s Genomics Shared Resource (GSR) for isolation of total cellular RNA and RNA-sequencing (RNA-Seq) analysis. Total cellular RNA was isolated from snap-frozen prostates using the TRIzol Reagent (Invitrogen, Waltham, MA, USA) followed by a clean-up with an RNeasy spin column (Qiagen, Hilden, Germany). Indexed, directional RNA-Seq libraries were prepared from 100 ng total RNA using the TruSeq Stranded mRNA (Illumina, San Diego, CA, USA) according to the manufacturer’s standard protocol. Two independent samples were run per group: wildtype mice (*Trp53^+/+^*), heterozygous mice (*Trp53^+/R270H^*), and homozygous mice (*Trp53^R270H/R270H^*). Subsequently, libraries were combined for multiplex sequencing on an Illumina HiSeq 4000 System (2 × 100 bp, paired-end; ≥30 × 10^6^ reads per sample). A HISAT-Cufflinks workflow was utilized for spliced alignment of the sequence reads (FASTQ format) to the reference genome assembly (December 2011, GRCm38/mm10), transcript assembly, quantitation, and differential expression analysis [34,35]. Gene-level FPKM expression values were used for downstream statistical analyses. Principal component analysis (PCA), differential expression analyses, hierarchical clustering, and heatmap visualization were performed with the GeneSpring GX (Agilent Technologies, Inc., Santa Clara, CA, USA) and Partek Flow (Partek, Inc, Chesterfield, MO, USA) software packages.

## 3. Results

### 3.1. B6.129S4-Trp53^tm3.1Tyj^/J Mice Develop PIN as Early as 3 Months of Age

**Figure 1:**
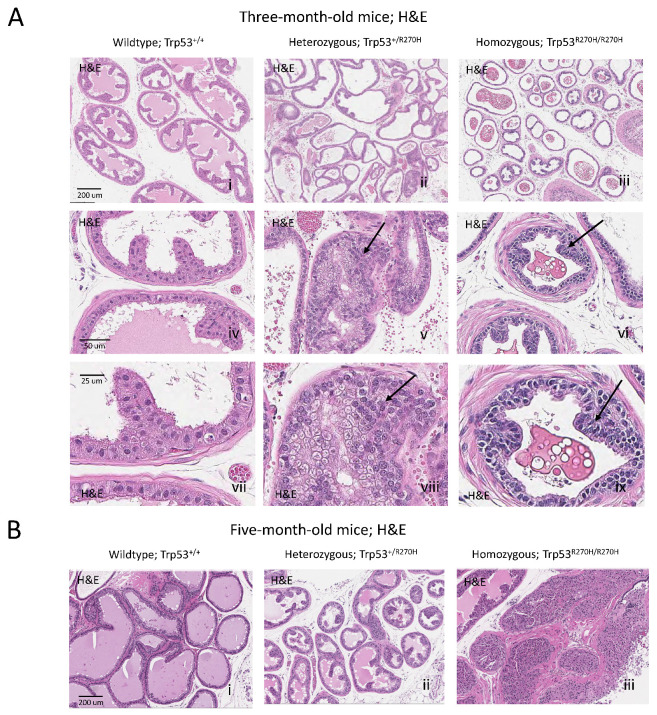
**The *Trp53^R270H^* mutant allele promotes the development of prostatic intraepithelial neoplasia (PIN).** At ~3 months of age, areas of metaplasia and atypical hyperplasia (arrows) were observed in heterozygous mice (*Trp53^+/R270H^*; **A**ii,v,viii) and grade 1 PIN lesions were observed in homozygous mice (*Trp53^R270H/R270H^*; **A**iii,vi,ix) but not wildtype mice (*Trp53^+/+^*; **A**i,iv,vii). PIN lesions (arrows) were observed in older *Trp53^+/R270H^* and *Trp53^R270H/R270H^*; mice (~5 months of age, **B**ii,iii, respectively). At ~5 months of age, grade 1 and grade 3–4 PIN lesions were observed in heterozygous and homozygous mice, respectively (**B**i,ii). PIN lesions were not observed in *Trp53^+/+^* (wildtype) mice at either time point (**A**i,**B**i). The combined data further confirm that the *Trp53^R270H^* mutation can drive initiation of prostate carcinogenesis. Please note that a representative scale bar is included in the first image for each horizontal panel.

Prostatic intraepithelial neoplasia (PIN) lesions were observed in B6.129S4-Trp53^tm3.1Tyj^/J mice that were homozygous (Trp53^R270H/R270H^) or heterozygous (Trp53^R270H/+^) for the *Trp53 R270H* mutation (Figure 1A), indicating that the p53 R270H mutation can drive CaP initiation in this setting. At 3 months of age, areas of metaplasia and atypical hyperplasia were observed in heterozygous mice, and grade one PIN lesions were observed in homozygous mice. At 5 months of age, heterozygous mice had developed grade one PIN lesions and homozygous mice had developed grade three to four PIN lesions. No metaplasia, atypical hyperplasia, or PIN lesions were observed in wildtype mice at either the 3 or 5 month time points. 

The ability of the p53 R270H mutation to drive CaP initiation in this setting, and the timing of PIN lesion incidence and PIN lesion grade aligns with what was observed by our group in a different *Trp53 R270H* GEM (*FVB.129S4 (Trp53^tm3Tyj^); FVB.129S (Nkx3-1^tm3(cre)Mms^)).* In our prior study, pre-PIN atypia and grade one PIN lesions were observed in homozygous mice as early as 5 weeks (note that this time point was not assessed in the current study) and grade three to four PIN lesions were observed at 6 months of age (compared to 5 months in the current model). In heterozygous mice, grade two PIN lesions were observed at 6 months of age (grade one PIN lesions were observed at 5 months in the current model). 

Our finding that the *Trp53 R270H* mutation can drive CaP initiation in two independent GEMs, and the similarities in the timing of PIN incidence and PIN grade between the GEM strongly supports a causative role for this mutation in driving prostate carcinogenesis. Note that PIN grading was performed as described by Park et al. for both studies [33].

### 3.2. Exposure to 5 Gy Whole Body Irradiation Stabilizes p53 Expression in the Majority of Mouse Prostate Cells

We demonstrate that exposure to 5 Gy irradiation stabilized p53 in all three groups of mice (*Trp53^+/+^* (wildtype), *Trp53^+/R270H^* (heterozygous), and *Trp53^R270H/R270H^* (homozygous for the *Trp53* mutation)); IHC analysis showed that more than 90% of prostate cells were p53-positive 6 h post-irradiation (Supplementary Figure S1). This is important because increased stabilization of p53 equates to increased transcriptional activity of p53 [36,37,38], and so irradiating mice helped enhance our ability to identify the differential expression of p53 target genes between the three groups, i.e., enhanced our ability to determine the impact of the *Trp53 R270H* mutation on the expression levels of these target genes. Cellular levels of p53 are usually kept very low via MDM2-mediated ubiquitination and subsequent proteasomal degradation, and irradiation is one of many environmental triggers that causes the inhibition of p53 degradation, and thereby causes p53 stabilization and increased transcriptional activity [36,37,38]. It is noteworthy that the 5 Gy dosage and 6 h time point were chosen for this study based on data from Levine et al.; the Levine lab demonstrated that 5 Gy whole-body ionizing irradiation causes stabilization of p53 in mouse spleen, heart, lung, kidney, liver, and skin at the 6 h post-irradiation time point [39]. While they did not look at p53 levels in the prostate, the prostate is well known to be a radiosensitive tissue and so we expected to also see p53 stabilization in the prostate at this time point. Our IHC analysis confirmed this to the case.

### 3.3. The Trp53 R270H Mutant Is Able to Alter Gene Expression in Mouse Prostate Cells, and in Many Cases Gene Expression Levels Correlate with Trp53 R270H Gene Dosage

**Figure 2:**
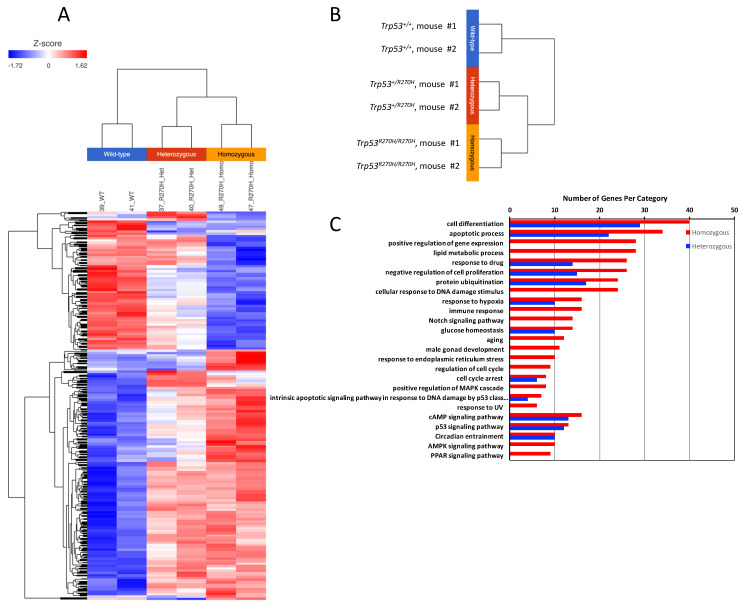
**RNA-seq analysis demonstrates that presence of the *Trp53^R270H^* mutant allele is able to alter the transcriptional response of prostate cells to 5 Gy radiation**. A distinct difference in gene expression patterns was observed between wildtype mice (*Trp53^+/+^*) versus heterozygous mice (*Trp53^+/R270H^*) and between wildtype mice (*Trp53^+/+^*) versus homozygous mice (*Trp53^R270H/R270H^*); heatmap (**A**), hierarchical clustering (**B**), and gene ontology (**C**) analyses. Differences between heterozygous and homozygous mice were also observed.

**Figure 3:**
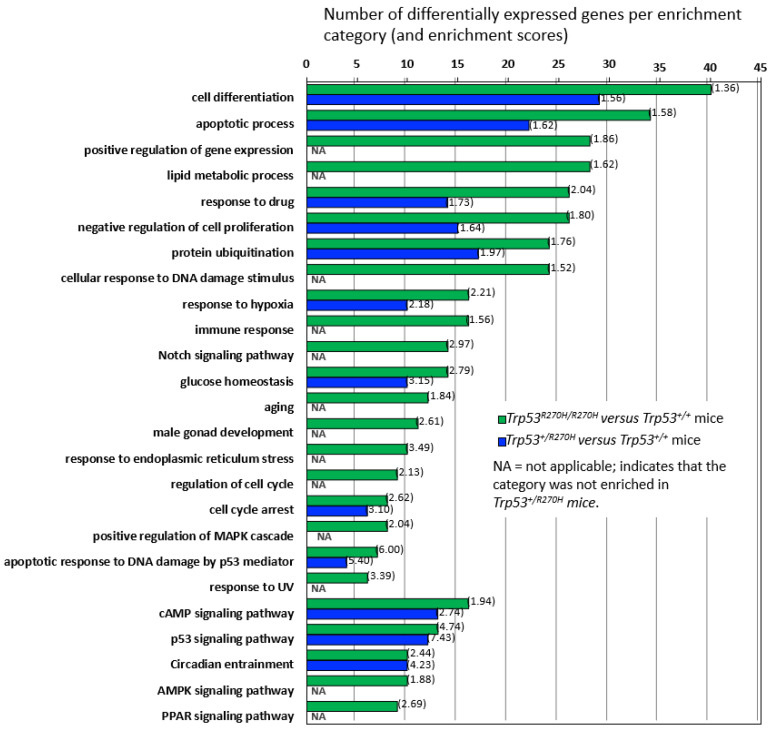
**Gene enrichment analysis and gene dosage effects.** Comparison of differential gene expression in *Trp53 R270H* homozygous versus wildtype mice and in *Trp53 R270H* heterozygous versus wildtype mice by gene enrichment category demonstrates that gene dosage differences exist for many, but not all of the categories. Gene enrichment scores are shown in brackets.

Three groups of mice (*Trp53^+/+^* (wildtype), *Trp53^+/R270H^* (heterozygous), and *Trp53^R270H/R270H^* (homozygous for the *Trp53* mutation)) were assessed as part of the RNA sequencing studies. Mice were exposed to 5 Gy irradiation and prostates were harvested for RNA extraction 6 h post-irradiation, i.e., at the time point that we had observed more than 90% IHC positivity for p53 for all three groups of mice (Supplementary Figure S1). Genes exhibiting differential expression (ANOVA, *p* < 0.05, +/−1.5-fold-change cut-off) across the three groups of mice were determined, and hierarchical clustering and heatmap visualization of the differentially expressed genes (DEGs) demonstrated that the mice within each group had very similar gene expression (Figure 2A,B). Assessment of the normalized expression values confirmed this (selected genes Table 1 and Table 2, entire dataset can be observed via this link; GEO accession GSE130440, https://www.ncbi.nlm.nih.gov/geo/query/acc.cgi?acc=GSE130440 accessed on 13 December 2021). The similarity of the RNA-Seq profiles within each group of mice indicate that our model is robust and gives confidence in the RNA-seq data produced. Statistically significant differences in gene expression between the three groups of mice were observed for 664 genes. *Trp53 R270H* gene dosage affects were observed for many genes, i.e., a further increase or decrease in gene expression relative to the wild type controls was observed in homozygous mice when compared to heterozygous mice (Table 1 and Table 2, Figure 3). This was not the case for all genes; in some instances, gene expression changed by a similar amount in both the *Trp53 R270H* heterozygous and homozygous mice relative to the wildtype mice, or only changed in the homozygous mice. Gene ontology analyses of these DEGs revealed a significant enrichment of genes belonging to the cell differentiation, apoptotic process, and cell proliferation categories (Figure 2C). Although the expression levels of DEGs were not always reflective of *Trp53* genotype status, there was a strong concordance with regard to function, in that *Trp53 R270H* homozygous mice typically exhibited a higher number of DEGs in enriched GO categories when compared to that of heterozygous mice. For example, for the ‘apoptotic process’ category comparison of *Trp53 R270H* homozygous versus wildtype mice, we identified 34 differentially expressed genes, while the comparison of *Trp53 R270H* heterozygous versus wildtype mice identified only 22 differentially expressed genes. Additionally, in the ‘positive regulation of gene expression’ category, DEGs were only observed in *Trp53 R270H* homozygous versus wildtype mice (28 differentially expressed genes), not *Trp53 R270H* heterozygous versus wildtype mice.

### 3.4. The Trp53 R270H Mutant Impacts Expression Levels of Known LOF and GOF Genes, as Well as Known CaP-Related Genes

Comparison of our list of differentially expressed genes with a previously published list of validated potential transcriptional targets of wildtype p53 (122 genes) that included targets validated by three different types of assay, including ChIP assay [23]) determined that 16 of the 122 validated targets of wildtype p53 were differentially expressed in our model (13%); *Acta1*, *Bax*, *Bbc3*, *Cav1*, *Ccng1*, *Cdkn1a*, *Ddit4*, *Gdf15*, *Hsp90ab1*, *Mdm2*, *Nos3*, *Ppm1j*, *Prkab1*, *Ptk2b*, *Tap1*, and *Tnfrsf10b* (Table 1). Expression of nine of these genes (*Bax*, *Bbc3*, *Ccng1*, *Cdkn1a*, *Ddit4*, *Gdf15*, *Mdm2*, *Tap1*, *Tnfrsf10b*) was decreased in heterozygous mice and *Trp53 R270H* gene dosage affects were observed for 7 of the 16 differentially expressed genes; *Bax*, *Bbc3*, *Ccng1*, *Cdkn1a*, *Ddit4*, *Gdf15*, and *Mdm2*. The fact that some but not all genes showed dosage effects, and that the impact of the mutant on gene expression varied between genes, can be interpreted as meaning that the *p53 R270H* mutant, a mutation which is located in the DNA binding domain of p53, is able to bind with different affinities to different gene promoters and that the interaction can be dose dependent. 

Comparison of our list of differentially expressed genes with a list of known *Trp53* GOF genes (total of 28 genes, [22]) identified two *Trp53* GOF genes that are differentially expressed in our model (2%); *Mgmt* and *Id4* (Table 2). Gene dosage effects were observed for only one of these (*Mgmt*). Our list of differentially expressed genes was also compared with a list of genes that are differentially expressed in CaP patients and that are known to contribute to CaP progression [40,41]. This comparison identified only four genes; *Cdkn1a*, *Kras*, *Raf1*, and *Cav1*. Gene dosage effects were observed for one of these, *Cdkn1a*. 

**Figure 4:**
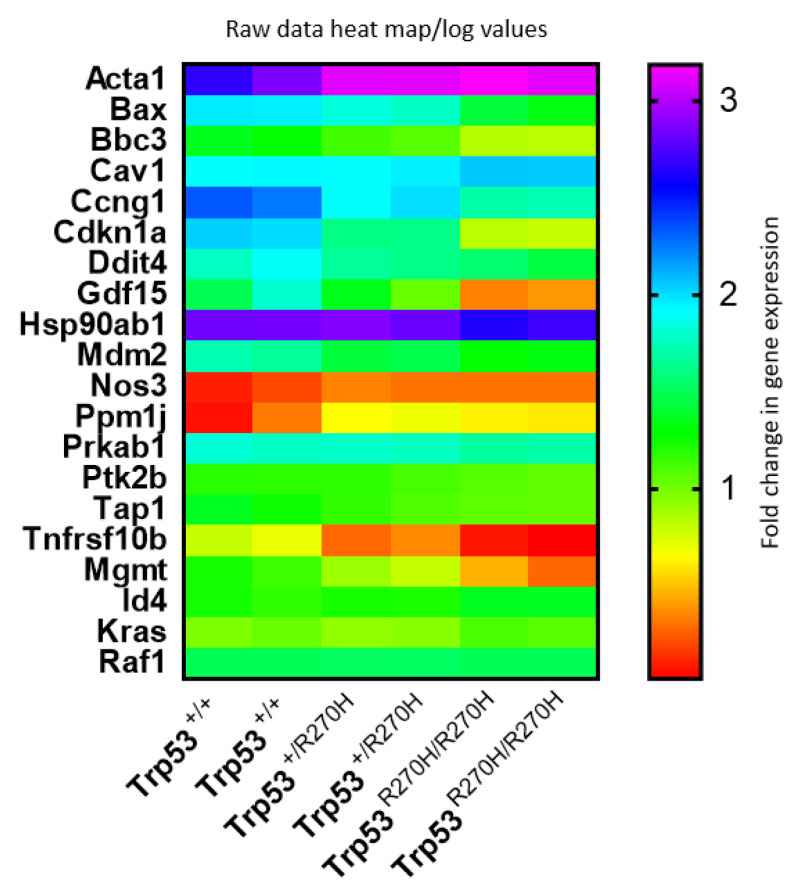
**Gene expression heat map.** Comparison of gene expression log values between the three genotypes and between differentially genes reveals that gene dosage effects exist for some, but not all genes, and that the relative impact of the p53 R270H mutant on gene expression varies widely between genes. This figure was generated using log transformed raw data for differentially expressed genes.

Heat map visualization of relative expression levels of genes listed in Table 1 and Table 2 (Figure 4) further confirms that gene dosage effects occur for many, but not all, of these genes, and that the relative impact of the p53 R270H mutant on gene expression varies widely between genes. This visualization also further confirmed that gene expression levels are very similar for each of the two mice in each group (*Trp53^+/+^* (wildtype), *Trp53^+/R270H^* (heterozygous), and *Trp53^R270H/R270H^* (homozygous for the *Trp53* mutation)).

### 3.5. The Trp53 R270H Mutation Causes Decreased Levels of Irradiation-Mediated Apoptosis but Does Not Appear to Impact Cell Proliferation 

**Figure 5:**
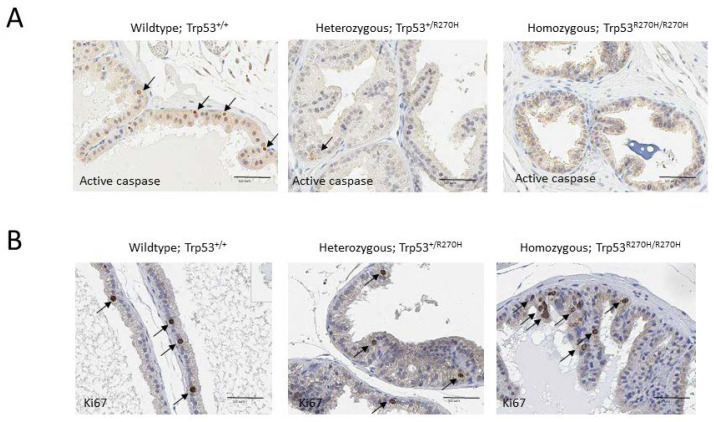
**Presence of the *Trp53^R270H^* mutant allele causes increased cell survival following irradiation but does not affect cell proliferation.** All mice were exposed to 5 Gy whole body irradiation 6 h prior to time of necropsy. IHC analyses of revealed that ~5% of cells in prostates from wildtype mice (*Trp53^+/+^*) expressed active caspase 3 (arrows), compared to only ~1% of cells in prostates from heterozygous mice (*Trp53^+/R270H^*) (**A**). Less than 1% of cells in prostates from homozygous mice (*Trp53^R270H/R270H^*) expressed active caspase 3 (**A**). These data indicate that the *Trp53^R270H^* mutant allele promotes survival of prostate cells. In contrast, Ki67 IHC revealed similar levels of cell proliferation (~2–5%) in all three genotypes (**B**).

IHC analyses were conducted to determine the impact of the *Trp53 R270H* mutation on irradiation-mediated apoptosis and cell proliferation. Irradiation is well known to impact both of these processes via both p53-dependent and p53-independent processes [42]. Apoptosis was assessed via activated caspase IHC, and cell proliferation was assessed via Ki67 IHC. Approximately 5% of cells in prostates from wildtype mice (*Trp53^+/+^*) expressed active caspase 3, compared to only ~1% of cells in prostates from heterozygous mice (*Trp53^+/R270H^*) (Figure 5A). Less than 1% of cells in prostates from homozygous mice (*Trp53^R270H/R270H^*) expressed active caspase 3. No differences in the number of cells expressing Ki67 were observed; approximately 2–5% of cells were Ki67 positive in all three groups of mice (Figure 5B). These data indicate that the *Trp53 R270H* mutant impacts apoptosis related-pathways to a greater extent when compared to cell proliferation-related pathways. This finding aligns with the gene ontology analysis of our RNA-seq data, which show more apoptosis-related genes were differentially expressed in the homozygous and heterozygous mice when compared to cell proliferation-related genes (Figure 2B).

## 4. Discussion

We previously demonstrated that the *Trp53 R270H* mutation can drive CaP initiation using the *FVB.129S4 (Trp53^tm3Tyj^); FVB.129S (Nkx3-1^tm3(cre)Mms^)* GEM [9]. We now validated this finding in a different GEM: B6.129S4-*Trp53*^tm3.1Tyj^/J. In both models, heterozygous mice developed PIN as early as 3 months. The main goal of the current study was to identify genes that may play a role in this process. RNA sequencing analysis of homozygous, heterozygous, and wildtype mice prostate samples identified a total of 1378 differentially expressed genes, demonstrating that the *Trp53 R270H* mutation has a strong impact on gene expression. Sixteen of these differentially expressed genes are known transcriptional targets of wildtype p53, while two of them are known to be p53 GOF genes. To our knowledge, this is the first study to document the impact of mutant p53 on prostate cell gene expression in a GEM which develops PIN. 

In addition to the GEM described in our previous and current study, there is only group who have generated a GEM to help determine the role of mutant p53 in driving CaP initiation. Elgavish et al. generated a GEM with prostate-specific expression of the *Tp53 R273H* mutant (a hotspot mutation in CaP patients) [43]. *Tp53 R273H* is the human equivalent of the mouse *Trp53 R270H* mutant. Their mutant mice developed grade three to four PINs at 52 weeks, and assessment of levels of apoptosis following castration revealed that apoptosis was reduced in mutant mice. These data align with ours; our mutant mice also developed PIN (albeit much earlier), and in our GEM the *Trp53 R270H* mutant caused decreased levels of apoptosis and decreased the expression of pro-apoptosis-related genes in response to irradiation. While other p53 GEMs exist, in these models, typically compound models, p53 expression has been silenced to mimic a loss of heterozygosity, a genetic alteration which is associated with late-stage CaP [44,45,46,47]. Generation and characterization of models which harbor p53 mutations, and placing focus on elucidating the role of p53 mutations in driving CaP incidence and progression is important because a significant number of CaP patients with early-stage disease harbor p53 mutations within their primary tumors, and p53 mutations have been found in human PIN lesions [48]. 

Two of the pro-apoptosis genes that we observed as being downregulated in our homozygous and heterozygous *Trp53 R270H* GEM are direct transcriptional targets of wildtype p53: Bax and Bbc3 (also known as Puma). Decreased Bax and/or Puma expression has been shown to be associated with increased pathological grade in CaP patients [49,50,51,52], and multiple CaP cell line and animal studies indicate that decreased expression of pro-apoptotic molecules can contribute to CaP progression and chemoresistance [53]. While a direct association between decreased Puma and/or Bax expression and CaP initiation has not been reported, it is well known that impaired cellular damage responses and dysregulation of apoptosis can contribute to the initiation of multiple cancer types, including CaP [54,55]. Several studies have demonstrated that environmental exposures, e.g., ingestion of environmental carcinogens or chronic inflammation, trigger apoptosis responses and are associated with a higher risk of CaP incidence [56,57]. While our mice were exposed to an extreme environmental trigger of apoptosis (5 Gy whole body irradiation), the combined data indicate it is possible that p53 mutant-mediated dysregulation of the apoptosis response to environmental carcinogens could potentially contribute to CaP initiation in some patients. 

Dysregulation of the cell cycle is well known to contribute to cancer initiation [58]. In our study, the expression of cell proliferation-related genes was also impacted by presence of the *Trp53 R270H* mutant, but to a lesser extent when compared to apoptosis-related genes; the cell proliferation gene category ranked sixth in the gene ontology analyses while the apoptosis-related gene category ranked second. Alterations in proliferative index (Ki67) were not observed, further supporting a lesser role for the dysregulation of cell proliferation in mediating p53 mutant-driven CaP initiation. The most notable cell cycle molecule to be impacted by the *Trp53 R270H* mutant was *Cdkn1a*. *Cdkn1a* is a transcriptional target of wildtype p53 and its protein product, p21, mediates G1 cell cycle arrest, primarily through inhibition of Cdk molecules, e.g., Cdk1 and 2 proteins [59]. While a direct link between CaP initiation and decreased p21 has not been reported, loss of p21 is associated with cancer initiation in other cancer types, e.g., liver cancer [60]. Interestingly, p21 expression is frequently increased in CaP patients and is associated with progression and worse outcomes [61,62]. This paradoxical effect has been hypothesized to result from the p21-mediated inhibition of apoptosis, and studies indicate that p21 can act as a tumor suppressor or oncogene, depending on cell type and context [63]. Whether p21 is acting as a tumor suppressor or oncogene in our model remains to be determined. 

Differential expression of two known GOF genes was also observed in our study; *Mgmt* and *Id4*. Mgmt (O6-methylguanine DNA methyltransferase) plays a key role in DNA repair; it removes DNA adducts that are added by alkylating agents, such as cisplatin and volatile *N*-nitroso compounds (found in high concentrations at rubber and metal factories), and thereby prevents the accumulation of mutations/genotoxicity [64,65]. In our GEM, the *Trp53 R270H* mutant causes the decreased expression of Mgmt, indicating that mutant mice will be more likely to accumulate DNA damage. It is possible that this mechanism contributes to mutant p53-initiated CaP. Expression levels of the other p53 GOF gene that our study identified, *Id4,* were elevated in mice that harbor the *Trp53 R270H* mutant. *Id4* encodes for a DNA-binding protein inhibitor that can regulate cell proliferation, apoptosis, and differentiation. Interestingly, Id4 has been shown to act upstream of wildtype p53; it acetylates wildtype p53 and thereby enhances its transcriptional activity [66]. Furthermore, studies have shown that increasing Id4 blocks the tumorigenicity of CaP cells [67]. Based on this information, it would appear that increased expression of Id4 should therefore inhibit prostate carcinogenesis, i.e., the opposite of what we would expect in this setting. 

The ability of the *Trp53 R270H* to drive breast and lung cancer initiation has been demonstrated in other GEM. Many consider the ability of the *Trp53 R270H* mutant to mediate cancer initiation is, in of itself, confirmation that this mutant can cause GOF, because p53-null mice do not develop breast or lung cancers [68,69,70]. In alignment with this, p53-null mice do not develop PIN or CaP in the absence of other genetic alterations [3]. Gene expression profiling studies have been performed in breast and lung cancer-specific p53-mutant GEMs and have also identified differential expression of apoptosis and cell cycle-related genes. Turrell et al. found that expression of *Bbc3*, *Ccng1*, and *Cdkn1a* were impacted by presence of the *Trp53 R270H* mutant in their lung cancer GEM [71], and Wijnhoven et al. showed that *Bbc3* expression is impacted in their breast cancer GEM [70]. These data support our findings and indicate that further analysis of the role of *Bbc3* and *Cdkn1a* in prostate carcinogenesis is warranted. To our knowledge, these breast and lung GEM studies did not identify dysregulation of p53 GOF genes. 

It is noteworthy that the *Trp53 R270H* mutant mediated dose-dependent effects for some, but not all, wildtype p53 transcriptional target genes. For example, gene dosage effects were observed for *Bax*, *Bbc3*, and *Cdkn1a*; ~1.5-fold, ~1.6-fold, and ~2-fold decreases in these genes were observed in heterozygous mice, respectively, while ~3.6-fold, ~2.9-fold, and 14-fold decreases were observed in homozygous mice. The majority of CaP patients with early disease who harbor *Tp53* mutations are heterozygous for the mutations and loss of heterozygosity (LOH) is typically only observed in advanced disease. Based on this and our gene expression profiling data, which show that apoptosis is dysregulated, it is likely that patients with LOH will have a worse response to cytotoxic chemotherapy. It is also noteworthy that only a small fraction of known transcriptional targets of wildtype p53 were significantly impacted by presence of the *Trp53 R270H* mutation; 16 out of 122 genes (13%). This has been shown to be the case in other studies and cancer types and it appears that the impact of mutant p53 is both cell type and context dependent. 

A major limitation of this study is the lack of validation of differentially expressed genes. This will be an important next step and focus will be placed on validating the differential expression of the genes discussed above. 

## 5. Conclusions

Our combined data confirm that the *Trp53 R270H* mutant can drive CaP initiation and indicate that the dysregulation of apoptosis plays a role in promoting mutant p53-mediated CaP initiation. Importantly, we identified several potential mediators of mutant p53-driven CaP, which thereby provide a starting point for future validation and mechanistic studies. Our molecular characterization of the *Trp53 R270H* GEM will help support its usage for diagnostic and prognostic biomarker development studies, as well as for pre-clinical studies of therapeutic agents. 

## Figures and Tables

**Table 1:** **Presence of the *Trp53^R270H^* mutant allele mediates alterations in the expression levels of validated transcriptional targets of wildtype p53.** The expression of 16 genes that are known transcriptional targets of wildtype p53 was impacted by the presence of the *Trp53^R270H^* mutant allele. It is noteworthy that allele dosage effects were observed for some genes; *Bax*, *Ccng1*, *Cdkn1a*, *Gdf15*, *Mdm2*. Data shown are fragments per kilobase of exon model per million mapped reads (FPKM).

Gene	*p* Value (ANOVA)	*Trp53^+/+^* (Mouse #1)	*Trp53^+/+^* (Mouse #2)	*Trp53^+/R270H^* (Mouse #1)	*Trp53^+/R270H^* (Mouse #2)	*Trp53^R270H/R270H^* (Mouse #1)	*Trp53^R270H/R270H^* (Mouse #2)
*Acta1*	0.03226382	459.72382	723.69403	1295.8693	1305.2902	1532.4598	1335.5698
*Bax*	0.00234206	92.46413	90.38328	67.5547	58.88641	26.7726	21.443594
*Bbc3*	0.00355776	22.755802	18.623598	13.3205	11.639098	6.8682103	6.6584005
*Cav1*	0.00357872	82.86131	84.6536	83.98589	89.3961	113.60498	110.93498
*Ccng1*	0.00381259	216.86192	178.55894	80.588196	99.719315	50.149597	54.22179
*Cdkn1a*	2.50 × 10^−5^	108.96001	100.64	40.4964	41.430794	6.70245	6.1654506
*Ddit4*	0.04823983	57.755894	79.54468	45.9636	41.706512	35.910603	27.632603
*Gdf15*	0.01252794	30.834797	61.762997	22.0756	10.823901	2.20235	2.4798
*Hsp90ab1*	0.03196126	669.3211	686.84717	753.2858	651.8237	433.094	504.98203
*Mdm2*	0.00440876	53.018593	45.430508	26.794598	29.479399	18.8087	21.066896
*Nos3*	0.04553094	1.25112	1.57297	2.18129	2.02219	2.01661	2.03199
*Ppm1j*	0.03864105	1.14598	2.0899	4.44729	4.88169	4.1659994	3.9569898
*Prkab1*	0.03862554	65.31939	59.280407	60.98009	58.167007	46.9706	50.6644
*Ptk2b*	0.04042444	15.3253975	15.081601	14.640602	12.964701	11.9638	11.0372
*Tap1*	0.04544611	22.922	17.592104	14.591501	12.070802	11.3092985	10.970899
*Tnfrsf10b*	0.00248734	6.25948	5.06814	1.88841	2.3068597	1.18625	1.04909

heterozygous (*Trp53^+/R270H^*) and/or or homozygous mice (*Trp53^R270H/R270H^*). A dose-dependent effect was observed for *Mgmt*. The expression of 4 genes which are associated with prostate cancer incidence and/or disease progression (*Cdkn1a*, *Kras*, *Raf1*, and *Cav1*) were impacted by the presence of the *Trp53^R270H^* allele. A gene dosage effect was observed for 1 of these, *Cdkn1a*. Data shown are fragments per kilobase of exon model per million mapped reads (FPKM).

**Table 2:** **The *Trp53^R270^* mutant allele mediates alterations in expression levels of validated transcriptional targets of the *Trp53^R270H^* allele as well as alterations in expression levels of genes associated with incidence of prostate cancer in patients.** Two known transcriptional targets of *Trp53^R270H^*, *Mgmt* and *Id4*, demonstrated differential expression in wildtype (*Trp53^+/+^*) versus heterozygous (*Trp53^+/R270H^*) and/or or homozygous mice (*Trp53^R270H/R270H^*). A dose-dependent effect was observed for *Mgmt*. The expression of 4 genes which are associated with prostate cancer incidence and/or disease progression (*Cdkn1a*, *Kras*, *Raf1*, and *Cav1*) were impacted by the presence of the *Trp53^R270H^* allele. A gene dosage effect was observed for 1 of these, *Cdkn1a*. Data shown are fragments per kilobase of exon model per million mapped reads (FPKM).

Gene	*p* Value (ANOVA)	*Trp53^+/+^* (Mouse #1)	*Trp53^+/+^* (Mouse #2)	*Trp53^+/R270H^* (Mouse #1)	*Trp53^+/R270H^* (Mouse #2)	*Trp53^R270H/^* *^R270H^* (Mouse #1)	*Trp53^R270H/^* *^R270H^* (Mouse #2)
Differentially expressed p53 gain-of-function genes							
*Mgmt*	0.00768433	17.0458	13.7068	7.800239	6.28203	2.90736	1.86971
*Id4*	0.02814294	17.548595	14.7531	17.234304	16.794598	22.8728	23.457294
Differentially expressed prostate cancer patient-related genes							
*Cdkn1a*	2.50 × 10^−5^	108.96001	100.64	40.4964	41.430794	6.70245	6.1654506
*Kras*	0.02647585	9.465839	10.611401	8.453041	8.89257	12.714198	11.6827
*Raf1*	0.00400151	30.975206	30.752499	32.999496	33.546703	31.184992	31.010395
*Cav1*	0.00357872	82.86131	84.6536	83.98589	89.3961	113.60498	110.93498

## Data Availability

RNA-seq data is available via the following link, GEO accession GSE130440, https://www.ncbi.nlm.nih.gov/geo/query/acc.cgi?acc=GSE130440, accessed on 13 December 2021).

## Supplementary Materials

The following supporting information can be downloaded at: https://www.mdpi.com/article/10.3390/biology11020218/s1. Supplementary Figure S1: Immunohistochemical analysis confirms p53 stabilization in all 3 groups of mice following exposure to 5 Gy irradiation.
