# Supplementary material for: Use of RNA-Seq and a Transgenic Mouse Model to Identify Genes Which May Contribute to Mutant p53-Driven Prostate Cancer Initiation
Source: Biology (Basel). 2022 Jan 29;11(2):218. doi: 10.3390/biology11020218 (PMC8869245; doi:10.3390/biology11020218)
Supplement: Supplementary file 1 [file biology-11-00218-s001.zip › biology-1491900-supplementary.pptx]

## Slide 1
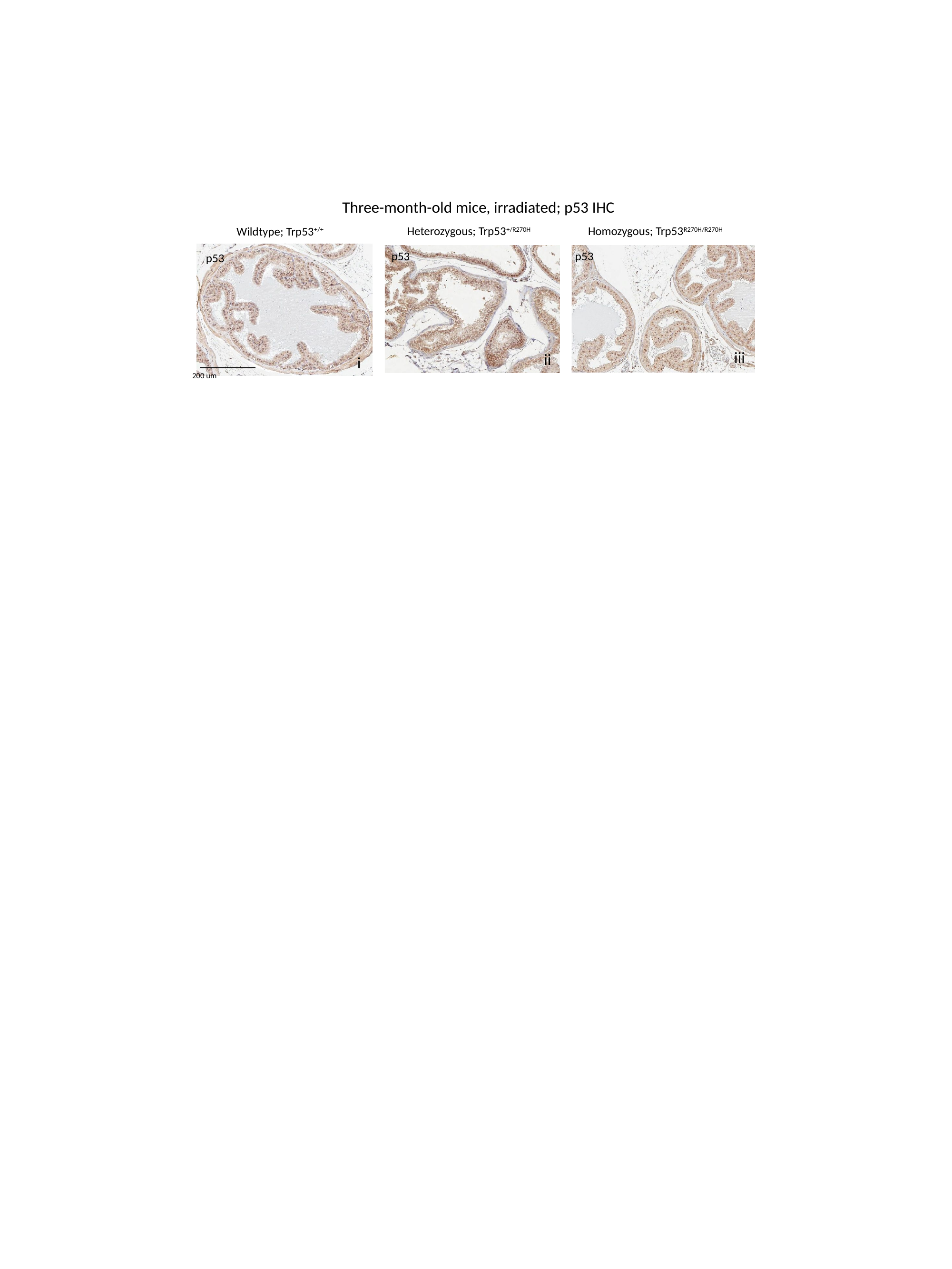

Three-month-old mice, irradiated; p53 IHC
Heterozygous; Trp53+/R270H
Homozygous; Trp53R270H/R270H
Wildtype; Trp53+/+
p53
p53
p53
iii
ii
i
200 um
